# Unravelling the diversity of glycoside hydrolase family 13 α-amylases from *Lactobacillus plantarum* WCFS1

**DOI:** 10.1186/s12934-019-1237-3

**Published:** 2019-10-26

**Authors:** Laura Plaza-Vinuesa, Oswaldo Hernandez-Hernandez, F. Javier Moreno, Blanca de las Rivas, Rosario Muñoz

**Affiliations:** 1 Instituto de Ciencia y Tecnología de Alimentos y Nutrición, ICTAN (CSIC), Juan de la Cierva 3, 28006 Madrid, Spain; 2 Instituto de Investigación en Ciencias de la Alimentación, CIAL (CSIC-UAM), CEI (UAM+CSIC), Nicolás Cabrera 9, 28049 Madrid, Spain

**Keywords:** α-Amylase, Lactic acid bacteria, Glycosyl hydrolase, GH13, Starch carbohydrates

## Abstract

**Background:**

α-Amylases specifically catalyse the hydrolysis of the internal α-1, 4-glucosidic linkages of starch. Glycoside hydrolase (GH) family 13 is the main α-amylase family in the carbohydrate-active database. *Lactobacillus plantarum* WCFS1 possesses eleven proteins included in GH13 family. Among these, proteins annotated as maltose-forming α-amylase (Lp_0179) and maltogenic α-amylase (Lp_2757) were included.

**Results:**

In this study, Lp_0179 and Lp_2757 *L. plantarum* α-amylases were structurally and biochemically characterized. Lp_2757 displayed structural features typical of GH13_20 subfamily which were absent in Lp_0179. Genes encoding Lp_0179 (Amy2) and Lp_2757 were cloned and overexpressed in *Escherichia coli* BL21(DE3). Purified proteins showed high hydrolytic activity on *p*NP-α-D-maltopyranoside, being the catalytic efficiency of Lp_0179 remarkably higher. In relation to the hydrolysis of starch-related carbohydrates, Lp_0179 only hydrolysed maltopentaose and dextrin, demonstrating that is an exotype glucan hydrolase. However, Lp_2757 was also able to hydrolyze cyclodextrins and other non-cyclic oligo- and polysaccharides, revealing a great preference towards α-1,4-linkages typical of maltogenic amylases.

**Conclusions:**

The substrate range as well as the biochemical properties exhibited by Lp_2757 maltogenic α-amylase suggest that this enzyme could be a very promising enzyme for the hydrolysis of α-1,4 glycosidic linkages present in a broad number of starch-carbohydrates, as well as for the investigation of an hypothetical transglucosylation activity under appropriate reaction conditions.

## Background

Starch is the most popular polysaccharide used as a food ingredient. It is a mixture of amylose, which is essentially composed only of α-1,4-linked glucose-polymers, and amylopectin, which is composed of α-1,4-linked glucose-polymers branched by α-1,6 linkages. α-Amylase specifically catalyse the hydrolysis of the internal α-1,4 glucosidic linkages of starch; in addition to its main reaction, α-amylase weakly catalyse α-1,4-transglycosylation [1]. Despite the fact that the catalytic action of any α-amylase should be, in principle, the same, different proteins may have evolved even within the same organism to possess the same catalytic activity as the α-amylase. In the sequence-based classification system of all carbohydrate-active enzymes (CAZy database, http://www.cazy.org) [2] α-amylase is one of the most frequently occurring glycoside hydrolase (GH). Family GH13 is known generally as the main α-amylase family. Overall, the α-amylases classified in family GH13 share 4-7 conserved sequence regions (CSRs) and catalytic machinery, and adopt the (α/β)_8_-barrel fold of the catalytic domain [3]. Within the family GH13, the α-amylase specificity is currently present in several subfamilies exhibiting a higher degree of sequence similarity to each other than to members of other GH13 subfamilies. Currently, 42 subfamilies of GH13 have been defined, but several sequences and characterized enzymes are not yet assigned to a subfamily [4]. Generally, GH13 subfamilies contain more than one reported activity. However, activities within each subfamily are closely related. An example is the assignment of different EC numbers for the same activity in subfamily GH13_20, which groups cyclomaltodextrinase (EC 3.2.1.54), maltogenic α-amylase (EC 3.2.1.133), and neopullulanase (EC 3.2.1.135), which are enzymes with strongly related (or even sometimes nearly identical) substrate and/or product specificities [5, 6]. These enzymes are distinguished from typical α-amylases (EC 3.2.1.1) by containing an N-terminal domain and exhibiting preferential substrate specificities for cyclomaltodextrins over starch [6].

*Lactobacillus plantarum* is a highly versatile lactic acid bacterial species found in many different ecological niches, such as vegetables, meat, fish, and dairy products, as well in the gastrointestinal tract. This ability to adapt to different environments and growth substrates is supported by its high genome size encoding a large variety of proteins, including those involved in carbohydrate utilization. Despite numerous genome sequences from *L. plantarum* are currently available, there is still limited information on the function of genes coding for α-amylases by their function on starch. It has turned out to be more appropriate to classify amylolytic enzymes based on similarities in their amino acid sequences and three-dimensional structures, and catalytic machineries, all reflecting evolutionary relatedness, rather than on specificity. Such approach, however, opens the door to enzymes, that are closely related in function, may be classified separately, but also to the fact that similar reactions can be catalysed by structurally different and, thus, evolutionary unrelated proteins [4]. The definitive approach to assign a specific molecular function to a predicted open reading frame is to biochemically characterize the corresponding protein. In this regard, the objective of this study was to determine the functional features of the putative Lp_2757 maltogenic α-amylase from *L. plantarum* WCFS1, through biochemical characterization of the recombinantly expressed protein. Moreover, as a maltose-forming α-amylase has been previously described from *L. plantarum* [7], a detailed comparison among the two *L. plantarum* amylolytic proteins has been also carried out.

## Results and discussion

### Sequence analysis and structural features of *L. plantarum* α-amylases

**Fig. 1:**
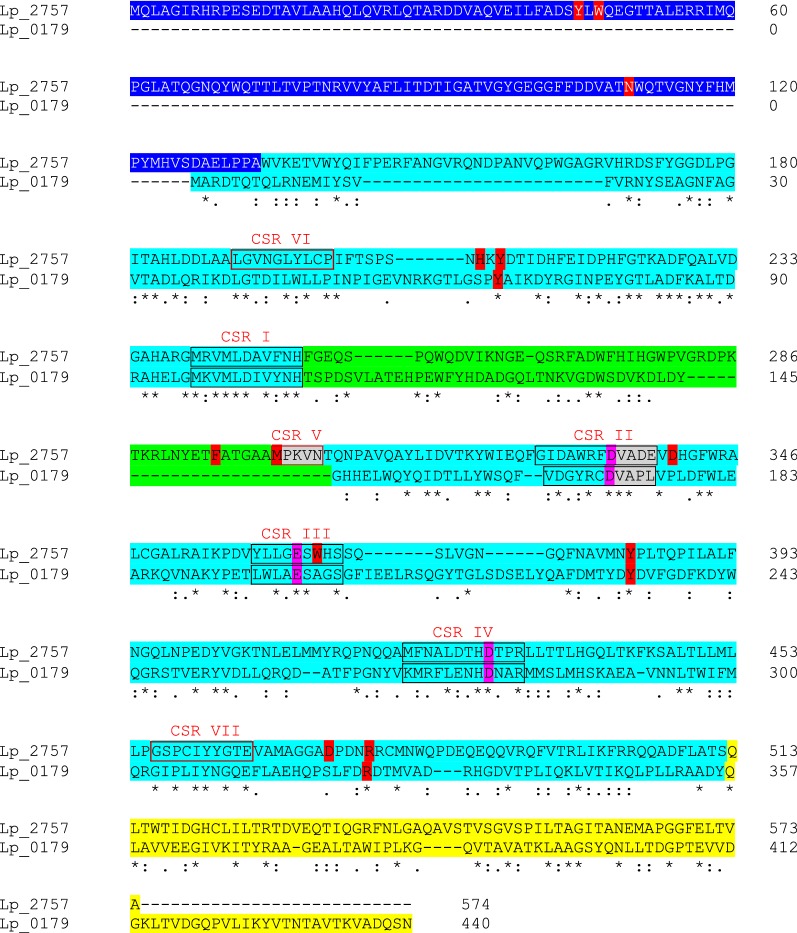
Comparison of amino acid sequences of Lp_2757 and Lp_0179 GH13 α-amylases from *L. plantarum* WCFS1. Aligment was done using the program ClustalOmega. Residues that are identical (*), conserved (:) or semiconserved (.) in both sequences are indicated. Dashes indicated gaps introduced to maximize similarities. The GH13 three domains are highlighted in blue (domain A), green (domain B), and yellow (domain C). The N-terminal domain of Lp_2757 (CBM34) is indicated in dark blue. The seven conserved sequence regions (CSR) found in GH13_20 subfamily α-amylases are also indicated. The residues of the GH13 catalytic triad (Asp-171, Glu-200, and Asp-227 in Lp_0179) are highlighted in pink colour. Conserved residues in amylolytic enzymes are highlighted in red (Lee et al., 2002). The conserved VAnE and MpKln motifs, in the CSR II and V respectively, are indicated in grey colour

As mentioned previously, family GH13 is known as the main α-amylase family in CAZy database. The GH13 polyspecificity results in the fact that the single membership to this family cannot be used for the prediction of gene function based on sequence alone [5]. *L. plantarum* WCFS1 possesses eleven proteins included in GH13 family. Among these proteins, only two proteins are annotated as “α-amylase”, Lp_0179 (Amy2, α-amylase) and Lp_2757 (maltogenic α-amylase). Amy2 from *L. plantarum* subsp. *plantarum* ST-III has been previously characterized and its maltose-forming α-amylase activity described [7]. Amy2 from this strain has an amino acid sequence identical to Lp_0179 (Amy2) from *L. plantarum* WCFS1 (data not shown). Lp_0179 is a 440 amino acid residues GH13 three-domain protein, harbouring the main catalytic (α/β)_8_-barrel domain (domain A) with a small domain B protruding out the barrel as a longer loop between the strand β3 and helix α3 and succeeded at the C-terminal end by domain C, adopting an antiparallel β-sandwich fold (Fig. 1). The domain of the (β/α)_8_-barrel is composed of eight inner parallel β-strands surrounded by eight α-helices and, because it was first recognized in the structure of triose-phosphate isomerase (TIM), is often called the TIM-barrel [4]. Lp_0179 possesses the four GH13 family conserved sequence regions (CSR) (CSR I, II, III, and IV) located at or near the C-termini of strands β3, β4, β5 and β7 of the catalytic (β/α)_8_-barrel domain and include the catalytic triad (Fig. 1, Additional file 1: Figure S1), The GH13 catalytic triad, consisting of Asp-171 (catalytic nucleophile), Glu-200 (proton donor), and Asp-277 (transition-state stabilizer), is present in Lp_0179 (Fig. 1). Throughout family GH13, sequence identity is extremely low and only the catalytic triad, plus the Arg-169, positioned two residues before the catalytic nucleophile, are generally conserved (Fig. 1; Additional file 1: Figure S1) [4].

Contrarily to Lp_0179, which has not yet been assigned to a subfamily, Lp_2757, the other *L. plantarum* GH13 α-amylase, is included in the GH13_20 subfamily. The members of this subfamily are distinguishable by the preferential substrate specificities for cyclomaltodextrins over starch, and by the presence of a N-terminal domain preceding the catalytic (α/β)_8_-barrel (Fig. 1, Additional file 2: Figure S2). Although the function of this N-terminal domain has still not been completely understood, such domain often acts as an anchor to starch in the catalytic reaction of the enzyme [8]. Typical starch-binding domains have also been classified within the CAZy database as the so-called “carbohydrate-binding modules” (CBM) families. CAZy database includes the Lp_2757 N-terminal domain in the family 34 (CBM34). In addition to this N-terminal domain, Lp_2757, as well as other GH13 α-amylases, possessed there additional CSR (CSR V, VI and VII), positioned near the C-terminus of domain B and at or near the C-termini of the barrel strands β2 and β8, and contained residues that may be used to distinguish the GH13 specificities from each other [4] (Fig. 1). GH13_20 α-amylases can be rather easily distinguished from other amylases on the basis of the conservation of Trp-47 and Phe-295 [6]. Moreover, Glu-338 is conserved in GH13_20 subfamily. This residue is proposed to play an important role in the binding of oligosaccharide acceptors [9]. Glu-338 is part of the conserved stretch VAnE succeeding the catalytic nucleophile in the CSR II (Fig. 1). Moreover, the GH13_20 subfamily is characterized by the sequence motif MpKln in their CSR V [10].

In spite of the fact that Lp_0179 and Lp_2757 are *L. plantarum* proteins annotated as α-amylases belonging to the GH13 family of glycosyl hydrolases, their amino acid sequence are only 26.4% identical, and are not closely related in terms of protein structure. Therefore, the recombinant production of both proteins and the subsequent analysis of their substrate specificity are warranted based on likely differences between their biochemical properties.

### Production, purification and biochemical characterization of recombinant *L. plantarum* α-amylases

**Fig. 2:**
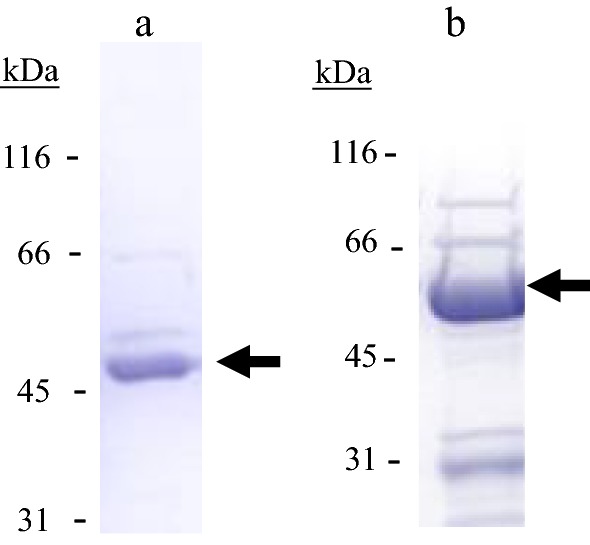
SDS-PAGE analysis of the purification of Lp_0179 (**a**) and Lp_2757 (**b**) GH13 α-amylases from *L. plantarum* WCFS1 after His affinity resin. The arrows indicated the overproduced and purified proteins. The gels were stained with Coomassie blue. Molecular mass markers are located at the left (SDS-PAGE Standards, Bio-Rad)

The only two proteins from the GH13 family of glycosyl hydrolases annotated as α-amylases, which exhibited substantial differences on their amino acid sequence, were recombinantly overproduced. The *lp_0179 (amy2)* and *lp_2757* genes were cloned into the pURI3-Cter expression vector by a ligation –free cloning strategy described previously [11]. The vector incorporates the DNA sequence encoding a C-terminal hexa-histidine tail to create His-tagged fusion enzyme for further purification step. The integrity of the constructs was confirmed by DNA sequencing. The *lp_0179* and *lp_2757* genes were expressed in *E. coli* under the control of an IPTG inducible promoter. Cell extracts were used to detect the presence of overproduced proteins by SDS-PAGE analysis. Whereas control cells containing the pURI3-Cter vector did not show protein overexpression, overproduced protein with an apparent molecular mass around 50 and 62 kDa for Lp_0179 and Lp_2757, respectively, were present in the intracellular soluble fraction of the cells (data not shown). Since the cloning strategy yielded His-tagged proteins, *L. plantarum* α-amylases were purified on an immobilized metal affinity chromatography (IMAC) resin. Recombinant proteins were eluted from the resin at 150 mM imidazole, and observed as an apparent band on SDS-PAGE (Fig. 2). The proteins were dialyzed against 50 mM phosphate buffer (pH 7) containing 300 mM NaCl.

Lp_0179 and Lp_2757 α-amylases are only 26.4% identical on their amino acid protein sequence, but taking into account that similar reactions can be catalysed by structurally different and unrelated proteins, the biochemical activity of pure recombinant proteins was analysed. A *p*NP-glycoside derivatives library to test the substrate range of glycosyl hydrolases was used. This library consisted of 25 commercially available *p*NP-glycosides to identify colorimetrically the ability of glycosyl hydrolases to liberate *p*-nitrophenolate at 420 nm. From the substrates assayed, Lp_0179 only hydrolyzed *p*NP-α-d-maltopyranoside, whereas Lp_2757 was able to hydrolyze *p*NP-α-d-maltopentaoside as well as *p*NP-α-d-maltopyranoside (86% of the activity on *p*NP-α-d-maltopentaoside). This result confirmed that both enzymes hydrolysed α-1,4-glucosidic bonds. However, the activity of Lp_0179 (LpMA) on *p*NP-α-d-maltopentaoside was previously analysed reporting that Lp_0179 hydrolyzed the α-1,4-glucosidic bonds of *p*NP-α-d-maltopentaoside, resulting in production of maltose and *p*NP-α-d-maltotriose [7]. This result could be in agreement with the results reported in our study, as Lp_0179 was unable to liberate *p*NP (detected colorimetrically) but able to hydrolyze one internal α-1,4-glucosidic bond to liberate *p*NP-α-d-maltotriose and maltose (detected by HPAEC and thin layer chromatography) [7].

**Fig. 3:**
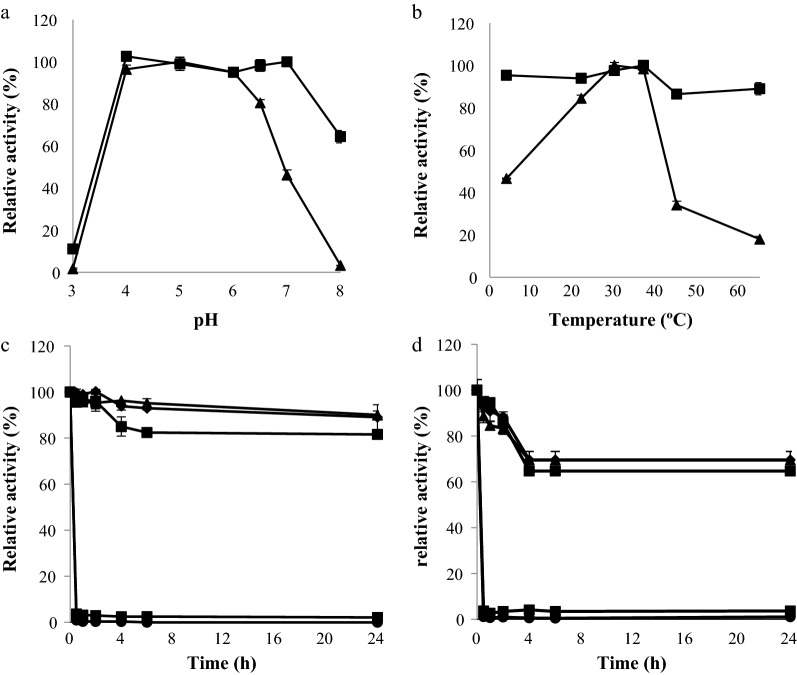
Biochemical properties of Lp_0179 and Lp_2757 α-amylases from *L. plantarum* WCFS1. **a** Relative activity of Lp_0179 (filled square) and Lp_2757 (filled triangle) versus pH. **b** Relative activity of Lp_0179 (filled square) and Lp_2757 (filled triangle) versus temperature. Thermal stability of Lp_0179 (**c**) and Lp_2757 (**b**) after preincubation at 22 °C (filled diamond), 30 °C (filled square), 37 °C (filled triangle), 45 °C (cross), and 65 °C (filled circle) in MOPS buffer (50 mM, pH 7) containing 20 mM NaCl and 1 mM DTT; at indicated times, aliquots were withdrawn, and analyzed as described in the Methods section. The experiments were done in triplicate. The mean value and the standard error are shown. The observed maximum activity was defined as 100%

As both α-amylases hydrolysed *p*NP-α-d-maltopyranoside, this substrate was chosen to characterize the biochemical properties of Lp_0179 and Lp_2757. Figure 3 showed a different behaviour of both α-amylases in relation to their optimal pH and temperature for activity. The optimal pH for activity is 4–7 in Lp_0179 and 4–6 for Lp_2757 (Fig. 3a). Lp_2757 presented its maximal activity at 30–37 °C, whereas Lp_0179 maintained a similar activity at the different temperatures assayed (Fig. 3b). Similarly to Lp_0179, a maltose-forming amylase from *L. plantarum* S21 strain, which is absent on WCFS1 strain, showed the same behaviour. *L. plantarum* S21 amylase exhibited more than 80% relative activity at pH values ranging from 5.0 to 6.5, and optimal temperature at all temperatures assayed [12].

It is interesting to note that when soluble starch was used as substrate, Lp_0179 showed a clear optimum temperature at 30 °C and more acidic optimal pH (pH 3) [7]. As explained before, the reason for the differences observed on Lp_0179 optimal pH and temperature could be partially due to the different glycosidic bond hydrolysed. The optimal reaction temperature on various substrates of an α-amylase from *Lactobacillus gasseri* ATCC 33323, 44% identical to Lp_2757, was determined to be 55 °C [13], higher than the optimal temperature observed for Lp_0179 and Lp_2757. The *L. gasseri* α-amylase showed its highest hydrolytic activity in 50 mM sodium acetate buffer at pH 5.0, as enzyme activities were dependent not only on pH but also on the type of buffer used [13]. α-Amylases from *Alicyclobacillus* sp. and *Bacillus subtilis* XL8 also exhibited high optimal temperature, 65 and 70 °C, respectively, and an acidic optimal pH for activity [14, 15].

In relation to their thermostability, both enzymes are heat-labile at temperature higher than 37 °C. However, at 37 °C or lower temperatures, Lp_0179 showed more than 80% activity after 24 h incubation whereas Lp_2757 showed only 60% activity (Fig. 3c, d). A mesophilic behaviour was observed in *Corallococcus* sp. α-amylase, as almost all activity remained after the enzyme was incubated 60 min at temperatures higher than 40 °C; however, a dramatic loss of enzymatic stability occurred at temperatures higher than 50 °C after 30 min of incubation [16]. Thermostable α-amylases were described in *Arthrobacter agilis* [17] and *Alicyclobacillus* [14], as they were highly active after prolonged incubations at 60 °C.

The effect of some metal ions and additives on *L. plantarum* α-amylase activity was studied (Table 1). Lp_0179 activity was significantly inhibited only by Hg^2+^ ions, and moderately inhibited by PMSF. None of the used additives increased significantly Lp_0179 hydrolytic activity. When starch was used as substrate, Co^2+^, Cu^2+^ and Fe^3+^ completely inhibited Lp_0179 activity [7]. Contrarily to Lp_0179, PMSF moderately increased Lp_2757 activity by an unknown mechanism. Cu^2+^ ions, as well as Hg^2+^ and Ni^2+^, significantly inhibited Lp_2757 hydrolytic activity on *p*NP-α-d-maltopyranoside. Both *L. plantarum* WCFS1 α-amylases were inhibited by Cu^2+^ ions, which also inhibited the activity of *L. plantarum* S21 [12], and *Corallococcus* sp. [16] α-amylases.

**Table 1:** Effect of additives on Lp_0179 and Lp_2757 α-amylase activity

Additives (1 mM)	Relative activity (%)	
	Lp_0179	Lp_2757
Control	100	100
KCl	93	90
HgCl_2_	6	2
CaCl_2_	98	81
MgCl_2_	96	58
ZnCl_2_	92	69
CuCl_2_	85	5
NiCl_2_	87	17
MnCl_2_	98	89
FeCl_2_	102	108
Tween 20	95	110
Tween 80	95	117
Triton X-100	96	120
SDS	86	59
Urea	100	95
DMSO	99	104
Cysteine	101	92
β-mercaptoethanol	99	107
EDTA	99	94
PMSF	68	134
DEPC	94	102

As Lp_2757 α-amylase also exhibited activity against *p*NP-α-d-maltopentaoside, their biochemical properties were also studied. Lp_2757 showed a similar behaviour against both *p*NP derivatives in relation to optimal pH and temperature, with the exception of a higher activity at pH 7.0 on *p*NP-α-D-maltopentaoside (data not shown). In a similar way to *p*NP-α-d-maltopyranoside, Cu^2+^, Hg^2+^ and Ni^2+^ also significantly inhibited Lp_2757 hydrolytic activity on *p*NP-α-d-maltopentaoside.

The kinetic parameters of Lp_0179 and Lp_2757 α-amylases were analysed using *p*NP-α-d-maltopyranoside as substrate at different concentrations. Despite that both α-amylases showed a similar *V*_max_ value, the *K*_m_ for Lp_2757 was approximately double than the *K*_m_ for Lp_0179. Therefore, the *E*_cat_ (*k*_cat_/*K*_m_) was remarkably higher for Lp_0179 than for Lp_2757. When the kinetic parameters of Lp_0179 were analysed using varied substrates, the catalytic efficiency was higher for soluble starch 2.06 (ml/mg) min^−1^ [7]. A higher catalytic efficiency on soluble starch was observed on the maltogenic amylase from *Corallococcus* sp. (16.91 (ml/mg) min^−1^) [16]. Among polysaccharides, the Lp_0179 catalytic efficiency was higher in amylose than in amylopectin, 1.44 and 0.45 (ml/mg) min^−1^, respectively [7]. The catalytic efficiencies showed by the maltose-forming α-amylase from *L. plantarum* S21 are higher, but followed the same preference on amylose [3.26 (ml/mg) min^−1^] and amylopectin [1.98 (ml/mg) min^−1^] [12].

Both *L. plantarum* α-amylases clearly exhibited structural, biochemical and kinetic differences, therefore a study of their activity on potential α-amylases carbohydrate-based substrates will allow to known unambiguously their substrate profile of both amylolytic proteins.

### Substrate profile of recombinant *L. plantarum* α-amylases

Cyclomaltodextrinase, neopullulanase, and maltogenic amylase share 40-86% sequence identity with each other. These enzymes are distinguished from typical α-amylases by containing a N-terminal domain and by exhibiting preferential substrate specificities for cyclomaltodextrins over starch, as well as high transglycosylation and hydrolytic activities. Concretely, these enzymes have shown to be capable of hydrolysing the potent inhibitor acarbose, and transfer the product to a sugar acceptor molecule [9]. A great deal of confusion exists regarding the features distinguishing the three groups of enzymes from one another. Although a different enzyme code has been assigned to each of the three different enzyme names, even a single differentiating enzymatic property has not been documented in the literature [6]. In consequence, it has been proposed that these enzymes should be classified under the same name and enzyme code to avoid confusion, as they are nearly the same enzymes in terms of their structures and catalytic properties and that they can be rather easily distinguished from other amylases.

**Fig. 4:**
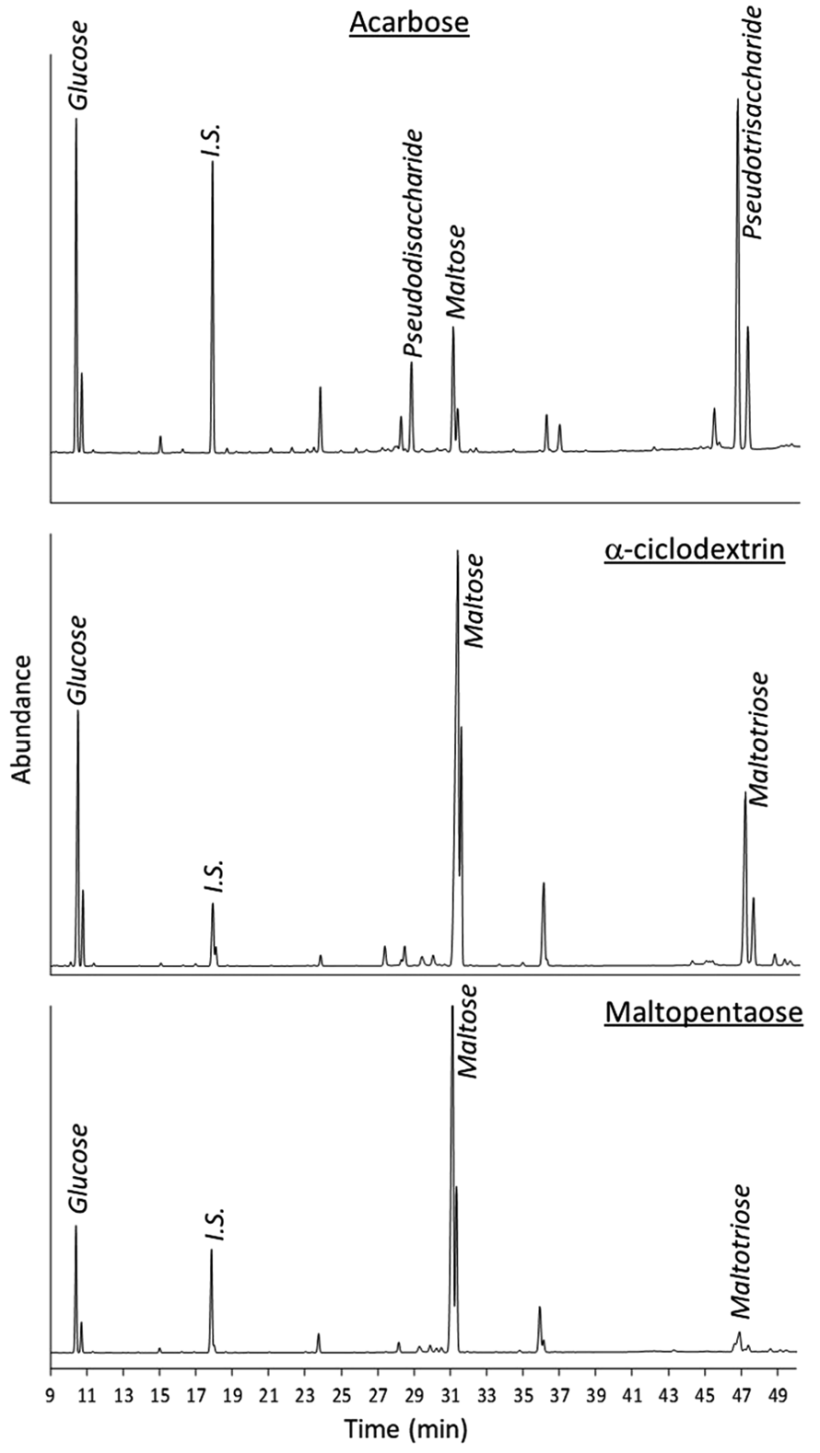
Chromatographic profiles (GC-FID) of the hydrolysis of acarbose, α-ciclodextrin and maltopentaose with Lp_2757 after 48 h

In order to known the substrate preference of Lp_0179 and Lp_2757 proteins, the potential hydrolysis of twelve starch-related carbohydrates were assayed and monitored by GC-FID for 48 h (Additional file 3: Table S1). Lp_2757 was highly efficient in hydrolysing ten out of twelve substrates, whereas the trisaccharide panose (α-d-Glc-(1 → 6)-α-d-Glc-(1 → 4)-d-Glc) was hydrolysed at a much lower extent and the polysaccharide dextran (a complex branched glucan predominantly consisting of α-1,6 glycosidic linkages) was not hydrolysed at all (Table 2). In all positive reactions, with the exception of acarbose, the main detected product from hydrolysis was maltose, which is in good agreement with the mechanism described for amylolytic enzymes belonging to the CAZy family_subfamily GH 13_20 (Fig. 4) [18]. The different behaviour observed for acarbose, a pseudotetrasaccharide and potent inhibitor of α-glucosidase and pancreatic α-amylase with antihyperglycemic activity, is due to its rather unusual structure which comprises a C-7 cyclitol moiety at the non-reducing end linked via nitrogen to a 4,6-dideoxyglucose which is in turn α-(1-4)-linked to maltose [19]. In this particular case, the main observed hydrolysis products were glucose and the corresponding pseudotrisaccharide, although maltose and the corresponding pseudodisaccharide were also found at lower amounts (Additional file 3: Table S1, Fig. 4). The hydrolysis of acarbose supports the classification of Lp_2757 as a maltogenic amylase as it is typical of this group of enzymes. On the other hand, the negligible and lack of hydrolysis of panose and dextran, respectively, revealed that Lp_2757 is essentially incapable of efficiently breaking α-1-6 glycosidic linkages. In contrast, the notable hydrolysis on a wide range of carbohydrate-based substrates demonstrated the great preference of Lp_2757 towards α-1,4-linkages present not only in cyclodextrins, confirming, thus, that this enzyme is a maltogenic amylase, but also in non-cyclic oligo- and polysaccharides such as dextrin, potato starch, amylose or amylopectin (Table 2). In any case, the release of maltose from dextrin and potato starch was remarkably higher than that from amylopectin and pullulan (Additional file 3: Table S1), likely due to the fact that the former are strictly composed by α-1,4-linkages, whereas amylopectin and pullulan are branched polysaccharides due to the occurrence of α-1,6-linkages every 25–30 glucose units in amylopectin and the 2:1 ratio of α-1, 4-glycosidic to α-1, 6-glycosidic bonds present in pullulan.

**Table 2:** Structural and biochemical properties of Lp_0179 and Lp_2757 α-amylases

	Lp_0179	Lp_2757
α-Amylase type	Maltose-forming α-amylase	Maltogenic amylase
Gene	*amy2*	–
Lenght (amino acid residues)	440	574
Mw (kDa)	49.9	64.4
*I*p	4.89	5.35
CAZy family	GH13	GH13
Subfamily	–	GH13_20
Intracellular	Yes	Yes
Presence of:		
Domains A, B and C	Yes	Yes
CSR I, II, III, and IV	Yes	Yes
CSR V	No	Yes
N-terminal domain	No	Yes
Catalytic triad (D-E-D)	Yes	Yes
MpKln motif	No	Yes
VAnE motif	No	Yes
Trp-47 (Lp_2757)	No	Yes
Phe-295 (Lp_2757)	No	Yes
Glu-338 (Lp_2757)	No	Yes
Hydrolysis of starchy carbohydrates		
Dextran (20 kDa)	No	No
α-Cyclodextrin	No	Yes
β-Cyclodextrin	No	Yes
γ-Cyclodextrin	No	Yes
Acarbose	No	Yes
Panose	No	Yes^a^
Amylopectin	No	Yes
Maltopentaose	Yes	Yes
Dextrin	Yes	Yes
Starch	No	Yes
Amylose	No	Yes
Pullulan	No	Yes
Liberation of *p*NP from		
*p*NP-α-d-maltopentaoside	No	Yes
*p*NP-α-d-maltopyranoside	Yes	Yes
* V*_max_ (μmol min^−1^)	0.017 ± 0.002	0.016 ± 0.001
* K*_m_ (mM)	0.32 ± 0.07	0.62 ± 0.12
* K*_cat_ (min^−1^)	231.36 ± 0.002	294.85 ± 0.001
* E*_cat_ (mM^−1^ min^−1^)	720.08 ± 152.85	480.76 ± 97.20
Temperature (optimum) (°C)	4–65	30–37
pH (optimum)	4–7	4–6
Activators	–	PMSF
Inhibitors	Hg^2+^	Hg^2+^, Cu^2+^, Ni^2+^

^a^Hydrolysis determined by the detection of very low levels of maltose and glucose

In contrast, the hydrolysis behaviour of Lp_0179 on the starch-related carbohydrates was quite different to that observed for Lp_2757, which is in line with the relevant dissimilarities found in the structural features between both *L. plantarum* α-amylases and that have been addressed above. Basically, Lp_0179 only hydrolysed two substrates, i.e. maltopentaose and dextrin, but it did not hydrolyze, for instance, α-cyclodextrin, β-cyclodextrin, and γ-cyclodextrin, which are substrates essentially resistant to hydrolysis by common α-amylases, corroborating that Lp_0179 is an exotype glucan hydrolase [7].

## Conclusion

This work has demonstrated that *L. plantarum* WCFS1 possesses, at least, two GH13 α-amylases, a maltose-forming α-amylase, Lp_0179, and a maltogenic α-amylase, Lp_2757. Table 2 summarizes all the structural features and biochemical properties, which were noticeably different, of these two *L. plantarum* α-amylases. Lp_2757 exhibited all the structural features typical of GH13_20 subfamily that were absent from Lp_0179. The differences between both α-amylases were confirmed when both proteins were recombinantly produced and biochemically characterized. Contrarily to maltose-forming Lp_0179 α-amylase, Lp_2757 maltogenic α-amylase was able to hydrolyze cyclodextrins as well as the inhibitor acarbose and other non-cyclic oligo- and polysaccharides dominated by the presence of α-1,4 glycosidic linkages. The presence of different α-amylases active on starch-related carbohydrates is consistent with this bacteria´s role as a member of the vegetable fermentation microbiota where these carbohydrates are abundant.

## Materials and methods

### Bacterial strains, plasmids, enzymes, and reagents

*Lactobacillus plantarum* WCFS1 was kindly provided by Prof. M. Kleerebezem (NIZO Food Research, The Netherlands). *Escherichia coli* DH10B and *E. coli* BL21 (DE3) were used as transformation and expression hosts in the pURI3-Cter vector [11]. The *E. coli* strains were cultured in Luria–Bertani (LB) medium at 37 °C and shaking at 140 rpm. When required, ampicillin was added to the medium at a concentration of 100 μg/mL.

### Cloning of two α-amylases from *L. plantarum*

Two genes encoding for putative α-amylases in *L. plantarum* WCFS1 were PCR-amplified by Advantage HD DNA polymerase (TaKaRa). Gene *lp_0179* (*amy2*) of 1320 bp encoding a protein annotated as α-amylase, maltodextrins and cyclodextrins (UniProtKB code: F9USZ1) was amplified by using primers 1750 (5´-*TAACTTTAAGAAGGAGATATACAT*tggcacgcgatacgcaaacgcaat) and 1751 (5´-*GCTATTAATGATGATGATGATG*atgattggactggtcagcaactttagt) (the nucleotides pairing the expression vector sequence are indicated in italics, and the nucleotides pairing the *lp_0179* gene sequence are written in lowercase letters). The gene *lp_2757* (1,722 bp) encoding a protein annotated as maltogenic α-amylase (UniProtKB code: F9URM8) was amplified by using primers 1800 (5´-*TAACTTTAAGAAGGAGATATACAT*atgcaactagctggaattaggcacc) and 1801 (*GCTATTAATGATGATGATGATGATG*agccacggttaattcaaagcctcc). Purified PCR products were inserted into the pURI3-Cter vector using a restriction enzyme- and ligation-free cloning strategy [11]. This vector produces recombinant proteins having a six-histidine affinity tag in their C-termini. *E. coli* DH10B cells were transformed, recombinant plasmids were isolated, and those containing the correct insert were identified by DNA sequencing, and then transformed into *E. coli* BL21 (DE3) cells for expression.

### Expression and purification of recombinant *L. plantarum* α-amylases

*Escherichia coli* BL21(DE3) was transformed with pURI3-Cter-0179 and pURI3-Cter-2757 recombinant plasmids. *E. coli* cells were grown in LB medium containing 100 μg/mL ampicillin until an optical density at 600 nm of 0.4 was reached and then induced by adding isopropyl-β-d-thiogalactopyranoside (IPTG) at 0.4 mM final concentration. Following induction, the cells were grown at 22 °C for 18 h and collected by centrifugation (8000*g*, 15 min, 4 °C). The cells were resuspended in phosphate buffer (50 mM, pH 7) containing 300 mM NaCl. Crude extracts were prepared by French press lysis of the cell suspension (three times at 1100 psi). The insoluble fraction of the lysate was removed by centrifugation at 47,000*g* for 40 min at 4 °C, and the supernatant was filtered through a 0.45 μm pore-size filter and then applied to a Talon Superflow resin (Clontech) equilibrated in phosphate buffer (50 mM, pH 7) containing 300 mM NaCl and 10 mM imidazole. The bound enzyme was eluted using 150 mM imidazole in the same buffer. The purity of the enzymes was determined by SDS-PAGE in Tris–glycine buffer. Fractions containing the His6-tagged Lp_0179 or Lp_2757 proteins were pooled and dialyzed against 50 mM sodium phosphate buffer, 300 mM NaCl, pH 7 at 4 °C using dialysis membranes (OrDial D35-MWCO 3500, Orange Scientific, Braine-l’Alleud, Belgium) of 3.5 kDa pore diameter. Dialyzed proteins were analyzed for glycosyl hydrolase activity.

### Enzyme assay

Hydrolase activity was determined by a spectrophotometric method using *p*-nitrophenyl α-d-maltopyranoside (Sigma-Aldrich) as the substrate. The rate of hydrolysis of *p*-nitrophenyl (*p*NP) α-d-maltopyranoside for 10 min at 30 °C was measured in 50 mM MOPS buffer pH 7.0 containing 20 mM NaCl and 1 mM DTT at 420 nm in a microplate spectrophotometer PowerWave HT (Bio-Tek, USA). The reaction was stopped by addition of sodium carbonate 1 M at pH 9.0. In order to carry out the reaction (75 µL), a stock solution of 25 mM of *p*-nitrophenyl-α-D-maltopyranoside was prepared in water and mixed with 50 mM MOPS buffer pH 7.0 containing 20 mM NaCl and 1 mM DTT to obtain a 1 mM substrate final concentration. Control reactions containing no enzyme were utilized to detect any spontaneous hydrolysis of the substrates tested. Enzyme assays were performed in triplicate.

### Substrate specificity

The substrate specificity of Lp_0179 and Lp_2757 *L. plantarum* α-amylases was determined by using a library of 25 *p*-nitrophenyl glycoside derivatives: *p*NP-α-d-galactopyranoside, *p*NP-α-d-glucopyranoside, *p*NP-α-d-manopyranoside, *p*NP-α-d-xylopyranoside, *p*NP-α-L-arabinofuranoside, *p*NP-α-L-arabinopyranoside, *p*NP-α-L-fucopyranoside, *p*NP-α-L-rhamnopyranoside, *p*NP-β-d-fucopyranoside, *p*NP-β-d-galactopyranoside, *p*NP-6-P-β-d-galactopyranoside*, p*NP-β-d-glucopyranoside, *p*NP-6-P-β-d-glucopyranoside*, p*NP-β-d-glucuronide, *p*NP-β-d-maltoside, *p*NP-β-d-mannopyranoside, *p*NP-β-d-ribofuranoside, *p*NP-β-d-xylopyranoside, *p*NP-β-L-fucopyranoside, *p*NP-N-acetyl-α-d-glucosaminide, *p*NP-N-acetyl-β-d-glucosaminide, *p*NP-α-d-maltopentaoside, *p*NP-α-d-maltopyranoside, *p*NP-β-d-cellobiose and *p*NP-β-d-lactopyranoside. Most of them were purchased from Sigma-Aldrich (St. Louis, MO, USA); *p*NP-6-P-β-d-galactopyranoside was purchased from BioGold (St. Louis, MO, USA), and *p*NP-6-P-β-d-glucopyranoside was synthesized by J. Cumella [20]. A stock solution of each *p*-nitrophenyl glycoside was prepared in water. The reaction mix consisted of 75 μL of mixed substrate and 4 μg of enzyme solution (in 50 mM MOPS buffer pH 7.0 containing 20 mM NaCl and 1 mM DTT). Reactions were carried out at 30 °C in a microplate spectrophotometer PowerWave HT (Bio-Tek, USA) as described above.

The enzymatic substrate profile of purified α-amylases was determined on the *p*NP-glycoside library to colorimetrically monitor hydrolysis measuring the liberation of *p*-nitrophenolate at 420 nm. The screening was performed in a 96-well Flat Bottom plate (Sarstedt) where each well contains a different substrate (1 mM). Amylase solution 4 μg (in 50 mM MOPS buffer pH 7.0 containing 20 mM NaCl and 1 mM DTT) was added to each well and reactions were followed by measuring the increase in absorbance at 420 nm for 10 min at 30 °C in a Synergy HT BioTek microplate spectrophotometer. Blanks without enzyme were carried out for each substrate and data were collected in triplicate and the average activities were quantified. Results are shown as means ± standard deviations.

In addition, the hydrolytic activity of Lp_0179 and Lp_2757 was also assayed by using putative α-amylase substrates such as potato starch, amylose, amylopectin, pullulan, dextrin 20 (dextrin from maize starch), dextran 20 kDa, α-cyclodextrin, β-cyclodextrin, γ-cyclodextrin, acarbose, d-panose and maltopentaose, all purchased from Sigma-Aldrich, (St.Louis, MO, USA). α-Amylases (20 µg) were incubated in 50 mM MOPS buffer pH 7.0 containing 20 mM NaCl, 1 mM DTT and 5% (w/v) of substrate with the exception of potato starch, amylose, amylopectin and pullulan that were added at 1% (w/v). The reactions were carried out at 30 °C for 24 or 48 h. GC-FID analyses were performed to determine the reaction products generated from the assayed substrates. The samples were analysed as trimethylsilylated oximes (TMSO) prepared following the method of Brobst and Lott (1966) [21]. Briefly, the oximes were formed by adding hydroxylamine chloride in pyridine (2.5% w/v) and silylated with hexamethyldisilazane and trifluoroacetic acid. The reaction mixtures supernatants were injected into an Agilent Technologies gas chromatograph (Mod 7890A) equipped with a flame ionization detector (FID) and a fused silica capillary column DB-5HT (5%-phenyl-methylpolysiloxane; 30 m × 0.25 mm × 0.10 µm) (Agilent) following the method described by Julio-Gonzalez et al. (2019) [22]. The quantification of the hydrolysis products was performed by the internal standard method using phenyl-β-glucoside and the corresponding standards calibration curves (glucose, maltose, maltotriose, maltotetraose and maltopentaose). Data acquisition and integration were performed using the Agilent OpenLab software.

### Kinetic parameters

The enzyme kinetics of Lp_0179 and Lp_2757 were studied using *p*NP-α-d-maltopyranoside as substrate. In addition, the Lp_2757 kinetics on *p*NP-α-d-maltopentaoside were also determined. Kinetic values of *K*_m_ and V_max_ were determined by nonlinear regression analysis fitting to Michaelis–Menten curves of formation rates of *p*NP as a function of the concentration of substrates from 0 to 10 mM (0, 0.1, 0.25, 0.35, 0.5, 1, 2, 3, 4, 5, 6, 7, 8, 9 and 10 mM).

### Effects of temperature, pH, and additives on α-amylase activity

The effects of pH and temperature on *p*NP-α-d-maltopyranoside hydrolytic activity of Lp_0179 and Lp_2757 were studied by using buffers of different pH ranging from 3 to 8. The buffers (50 mM) used were citrate buffer (pH 3), acetic acid-sodium acetate (pH 4–6), MOPS (pH 6.5 and 7) and Tris–HCl (pH 8). The optimal temperature was assayed by incubating purified α-amylases in 50 mM MOPS buffer pH 7.0 containing 20 mM NaCl, 1 mM DTT at different temperatures (4, 22, 30, 37, 45 and 65 °C). For temperature stability measurements, recombinant α-amylases were incubated in 50 mM MOPS buffer pH 7.0 containing 20 mM NaCl, 1 mM DTT at 22, 30, 37, 55 and 65 °C for 30 min and 1, 2, 4, 6, and 20 h. Aliquots were withdrawn at these incubation times to test the remaining activity at standard conditions. The non-heated enzymes were considered as control (100%). The analyses were performed in triplicate.

To study the effect of metals and ions on α-amylase activity, the enzymes were incubated in the presence of the different additives at a final concentration of 1 mM for 5 min at room temperature. Then, *p*NP-α-d-maltopyranoside was added, and the reaction mixture was incubated 10 min at 30 °C. The residual hydrolytic activity was measured after the incubation of purified enzymes with each additive. The analyzed additives were, KCl, CaCl_2_, HgCl_2_, ZnCl_2_, CuCl_2_, NiCl_2_, FeCl_2_, MnCl_2_, Triton-X-100, Tween 20, Tween 80, SDS, urea, DMSO, cysteine, β-mercaptoethanol, PMSF, DEPC and EDTA. Hydrolytic activity measured in the absence of any additive was taken as control (100%). Experiments were done in triplicate.

## Supplementary information

**Additional file 1: Figure S1.** Sequence alignment of the α-amylase representatives of thirteen different GH13 subfamilies with focus on the intermediary group with MPDLN as CSR V [10]. The sequences from GH13_4 (*Neisseria polysaccharea*, accession Q9ZEU2), GH13_16 (*Propionibacterium freudenreichii* subsp. *Shermanii*, A1XGB1), GH13_17 (*Apis mellifera*, Q25BT8), GH13_18 (*Bifidobacterium adolescentis*, Q84HQ2), GH13_20 (*Lactobacillus plantarum* WCFS1, F9URM8), GH13_21 (*Escherichia coli*, P21517), GH13_23 (*Xhantomonas campestris*, Q76LB0), GH_29 (*Bacillus subtilis*, P39795), GH13_30 (*Thermonospora curvata*, Q60027), GH13_31 (*Bacillus cereus*, P21332), GH13_34 (*Xenopus leavis*, Q7ZYR3), GH13_35 (*Xenopus leavis*, Q32NL8), GH13_36 (*Paenibacillus polymyxa* E681, E0RLH8) subfamilies and Lp_0179 (NI, not included in a subfamily, F9USZ) are represented. Aligment was done using the program ClustalOmega. Residues that are identical (*), conserved (:) or semiconserved (.) in all sequences are indicated. Dashes indicated gaps introduced to maximize similarities. The GH13 three domains are highlighted in blue (domain A), green (domain B), and yellow (domain C). The catalytic triad is highlighted in pink. The seven GH13 CSRs are boxed. Conserved residues in amylolytic enzymes are highlighted in red [6].

**Additional file 2: Figure S2.** Sequence alignment of the α-amylase family GH13_20 representatives from *Alicyclobacillus acidocaldarius* (AAC) (accession Q9WX32), *Thermoactinomyces vulgaris* (TVU) (Q08751), *Klebsiella oxytoca* (KOX) (Q48398), *Bacillus* sp. I-5 (BSP) (Q59226), *Lysinibacillus sphaericus* (LSP) (Q08341), *Anoxybacillus flavithermus* (AFL) (Q5BLZ6), *Geobacillus caldoxylosilyticus* (GCA) (CoLZ63), *Thermus* sp. IM6501 (O69007), *Bacillus stearothermophilus* (BST) (P38940), and *Lactobacillus plantarum* WCFS1 (LPL) (F9URM8). Aligment was done using the program ClustalOmega. Residues that are identical (*), conserved (:) or semiconserved (.) in all sequences are indicated. Dashes indicated gaps introduced to maximize similarities. The GH13 three domains are highlighted in blue (domain A), green (domain B), and yellow (domain C). The N-terminal domains are indicated in dark blue. The seven conserved sequence regions (CSR) found in GH13_20 subfamily α-amylases are also indicated. The residues of the GH13 catalytic triad (Asp-171, Glu-200, and Asp-227 in Lp_0179) are highlighted in pink colour. Conserved residues in amylolytic enzymes are highlighted in red [6]. The conserved VAnE and MPKLn motifs, in the CSR II and V respectively, are indicated in grey colour.

**Additional file 3: Table S1.** Concentrations (mg/mL) of the products after the enzymatic hydrolysis with Lp_2757.
