# Supplementary material for: Unravelling the diversity of glycoside hydrolase family 13 α-amylases from Lactobacillus plantarum WCFS1
Source: Microb Cell Fact. 2019 Oct 26;18:183. doi: 10.1186/s12934-019-1237-3 (PMC6815381; doi:10.1186/s12934-019-1237-3)
Supplement: Supplementary file 1 — Additional file 1: Figure S1. Sequence alignment of the α-amylase representatives of thirteen different GH13 subfamilies with focus on the intermediary group with MPDLN as CSR V [10]. The sequences from GH13_4 (Neisseria polysaccharea, accession Q9ZEU2), GH13_16 (Propionibacterium freudenreichii subsp. Shermanii, A1XGB1), GH13_17 (Apis mellifera, Q25BT8), GH13_18 (Bifidobacterium adolescentis, Q84HQ2), GH13_20 (Lactobacillus plantarum WCFS1, F9URM8), GH13_21 (Escherichia coli, P21517), GH13_23 (Xhantomonas campestris, Q76LB0), GH_29 (Bacillus subtilis, P39795), GH13_30 (Thermonospora curvata, Q60027), GH13_31 (Bacillus cereus, P21332), GH13_34 (Xenopus leavis, Q7ZYR3), GH13_35 (Xenopus leavis, Q32NL8), GH13_36 (Paenibacillus polymyxa E681, E0RLH8) subfamilies and Lp_0179 (NI, not included in a subfamily, F9USZ) are represented. Aligment was done using the program ClustalOmega. Residues that are identical (*), conserved (:) or semiconserved (.) in all sequences are indicated. Dashes indicated gaps introduced to maximize similarities. The GH13 three domains are highlighted in blue (domain A), green (domain B), and yellow (domain C). The catalytic triad is highlighted in pink. The seven GH13 CSRs are boxed. Conserved residues in amylolytic enzymes are highlighted in red [6]. [file 12934_2019_1237_MOESM1_ESM.pdf]

Figure S1

```
20      MQLAGIRHRPESEDTAVLAAHQLQVRLQTARDDVAQVEILFADSYLW---QEGTTALE-R 56
21      ----------------MLNAWHLPVPPFVKQS-K---DQL--LITLW---LTGEDPPQ-R 34
18      ------------------------------------------------------------ 0
NI      ------------------------------------------------------------ 0
34      -----MT----------------------------Q-DTALDMKDVELNELDQEKVPMAG 26
4       -----MLTP-------------------------TQ-QVGLILQYLK-TRILDIYTPEQR 28
36      ------------------------------------------------------------ 0
16      ------------------------------------------------------------ 0
29      ------------------------------------------------------------ 0
31      ------------------------------------------------------------ 0
30      ------------------------------------------------------------ 0
23      ------------------------------------------------------------ 0
17      ------------------------------------------------------------ 0
35      -----MEEEPNSVE--------------LKQKAGHE-NNGFISDHD--G--RDEDKPEKG 36

20      RIMQ-----------------------PGLATQGNQYWQTTLTVPTN--RVVYAFLITDT 91
21      IMLRT--EHDNEEMSVPMH------KQRSQPQPGVTAWRAAIDLSSGQPRRRYSFKL--- 83
18      ------------------------------------------------------------ 0
NI      ------------------------------------------------------------ 0
34      G-AGDSPNGGAEKNGV-VKVKLEDEDEKSQKFTGLSKEE--LLRVAGTPTWVR------- 75
4       AGI--EKSEDWRQFSRRMDTHFPKLMNELDSVYGNNEA---------------------- 64
36      ----------------------------------------------------M------- 1
16      ------MTSDQNM----TS-DQHSETPPIRALLGEDEER--TA----------------- 30
29      ------------------------------------------------------------ 0
31      ------------------------------------------------------------ 0
30      ------------------------------------------------------------ 0
23      ------------------------------------------------------------ 0
17      ------------------------------------------------------------ 0
35      KSRRNSKEGDAHIYSVRIN-EVPEDSPPLKPYAGMPKDV--LLQFSNRPCYRI------- 86

20      IGATVGYGEGGFFDDVATNWQTVGNYFHMPY---------MHVSD------AELPPAWVK 136
21      ------------LWHDRQRWFTPQGFSRMPPARL----EQFAVDV------PDIGPQWAA 121
18      ------------------------------------------------------------ 0
NI      -------------------------------------------------MARDTQT-QLR 10
34      ----VRWALLILFWLG------WAGMLAGAVVII----------VQAPRCRPLPAMEWWN 115
4       ----------------------LLPMLEMLLAQAWQSYSQRNSSLKDIDIARENNPDWIL 102
36      -------TRNKRLRRL------STAMLTVPMLTM-----FASGAMAEQELNGHKPPVSTG 43
16      ---------------D------ARAKLEMALSLT-----QPTGAVDP------HDTSWFE 58
29      --------------------------------------------MKT------EQTPWWK 10
31      -----------------------------------------------------MEKQWWK 7
30      ----------------------------MQQVPD-----SVHLTAPT------AHTHWWR 21
23      ----------------------------------------------M------SQTPWWR 8
17      -----------------------MKSLVVVVLLL-----AVGLGAGQ------NNKGWWK 26
35      -------AREIIFWLI------IVATLAI-IAAT-----IAIIAISP------KCLDWWQ 121

20      ET--VWYQIFPERFANGVRQNDPAN-------------VQPWGA---GRVHRDSFYGGDL 178
21      DQ--IFYQIFPDRFARSLPREAEQDHVYYHHAAGQEIILRDWDEPVTAQAGGSTFYGGDL 179
18      ----MKNKVQLITYADRL-------------------------------------GDGTI 19
NI      NE--MIYSVFVRNYS----------------------------------------EAGNF 28
34      KG--PLYQVGDPA--TFQED-----------------------------------GAGNI 136
4       SNKQVGGVCYVDL------------------------------------------FAGDL 120
36      SG--VFYEIYINSFYDSNGD-----------------------------------GHGDL 66
16      TA--VFYEVMVRSFADSSGD-----------------------------------GIGDL 81
29      KA--VVYQIYPKSFNDTTGN-----------------------------------GVGDL 33
```

```
31   ES--VVYQIYPRSFMDSNGD-----------------------------------GIGDL 30
30   HA--VIYQIYVRSFADSNGD-----------------------------------GEGDL 44
23   GA--VIYQIYPRSFLDSNGD-----------------------------------GVGDL 31
17   NA--IFYQVYPRSFMDSNSD-----------------------------------GIGDL 49
35   RS--PMYQVYPKSFKDSNND-----------------------------------GSGDL 144
                                                              *  :

                          CSR VI
                            β2
20   PGITAHLDDLA--------ALGVNGLYLCPIFTSPS-------NHKYDTIDHFEIDPHFG 223
21   DGISEKLPYLK--------KLGVTALYLNPVFKAPS-------VHKYDTEDYRHVDPQFG 224
18   KSMTDILRTRF--------DGVYDGVHILPFFTPFD-----GADAGFDPIDHTKVDERLG 66
NI   AGVTADLQRIK--------DLGTDILWLLPINPIGEVNRKGTLGSPYAIKDYRGINPEYG 80
34   QSIEKRIESLS--------SLKVKGLIIGPIHVTKK--------DQIGETQLTDIDPNYG 180
4    KGLKDKIPYFQ--------ELGLTYLHLMPLFKCPE----GKSDGGYAVSSYRDVNPALG 168
36   KGITQKLDYLNDGKSHSGKDLQVSGLWLMPLNPSPS------Y-HKYDVTDYYQVDPQYG 119
16   AGLTEHLDYLQ--------WLGVDCLWLPPFFASPM------TDGGYDVADYRAISPDLG 127
29   NGIIEKLDYLK--------TLQVDVLWLTPIYDSPQ------HDNGYDIRDYYSIYPEYG 79
31   RGIISKLDYLK--------ELGIDVIWLSPVYESPN------DDNGYDISDYCKIMNEFG 76
30   NGIRERLPALV--------SLGVDAIWLTPFYVSPL------ADGGYDVADYRDVDPRFG 90
23   PGIIAKLDYIS--------GLGVDAIWISPFFKSPM------ADFGYDISDYRAVDPLFG 77
17   KGIKDKLSHFI--------ESGITAIWLSPINRSPM------VDFGYDISDFKDVDPIFG 95
35   KGVQEKIDHFL--------YLNIKNVWVAPFYKSSL------KDFNYAVDDFMEVDPTFG 190
      .:   :                  : : *.                .   :    *

                       CSR I
                         β3
20   TKADFQALVDGAHARGMRVMLDAVFNHFGEQS------PQWQDVIKNG-------EQSRF 270
21   GDGALLRLRHNTQQLGMRLVLDGVFNHSGDSH------A-WFDRHNRGTGGACHNPESPW 277
18   SWDDVAELSK-----THNIMVDAIVNHMSWES-------KQFQDVLAK------GEESEY 108
NI   TLADFKALTDRAHELGMKVMLDIVYNHTSPDSVLATEHPEWFYHDADG------------ 128
34   TKEQFASLLEAARKKSIQIILDLTPNYHS-EK-------SWFEKTERE------------ 220
4    TIGDLREVIAALHEAGISAVVDFIFNHTSNEH-------EWAQRCAAG-------DPLFD 214
36   SLNDFRTLMKEAHRKGVKVIMDLIINHSSSDH-------PWFKEASAN-------PQSKY 165
16   TLSEFGDFIDKAHDRGIRIIIDFVMNHTSDHH-------PWFEESRRH-------PDGPY 173
29   TMEDFERLVSEAHKRDLKVVMDLVVNHTSTEH-------KWFREAISS-------IDSPY 125
31   TMEDWDELLHEMHERNMKLMMDLVVNHTSDEH-------NWFIESRKS-------KDNKY 122
30   TLADFDALLATAHDLGLRVIIDIVPNHTSSAH-------RWFRDALAA------GPGSPE 137
23   SLADFDRLLEKAHGLGLKVMIDQVLSHTSIAH-------AWFQESRQD-------RSNPK 123
17   TIKDLEDLTAEAKKQNLKVILDLVPNHTSDQH-------KWFQMSINNT---NNNNTNKY 145
35   TMADFESMISAMHDKGLKLIIDLIPNHTSNKH-------KWFQLSRN--------RTGKY 235
             .           ::*   .: .

20   ADWFH-----IHGWPVGRDP---------------------------KTKRLNYETFAT 297
21   RDWYS-----FSDDGT---------------------------------ALDWL---G 294
18   YPMFLTMSSVFPNGATEEDL----A--------GIYRPRPGLPFTHYKFAGKTRLVWVSF 156
NI   -------------QLTNKV---------------------------------------GD 136
34   -------------SI--------------------------VD----------------- 224
4    NFYYI-----FPDRRMPDQYDRTLREIFPD-----QHPG-GFS-----QLEDGRWVWTTF 258
36   HDYYV-----WADENTDLEE-------------KGSWGQQIWHKNP----NGEGYFYGTF 203
16   GDFYV-----WRDNP---DG------YPDARIIFIDTETSNWTFDP----VRGQYFWHRF 215
29   RDFYI-----WKKPQ--ENG------SVPT-NWESKFGGSAWELDE----ASGQYYLHLF 167
31   RDYYI-----WR-PG--KEG------KEPN-NWGAAFSGSAWQYDE----MTDEYYLHLF 163
30   RDRYV-----FRPGRGENGE------LPPN-NWQSIFGGPAWTRVTEPDGTPGEWYLHLF 185
23   ADWYV-----WADPR--EDG------TPPN-NWLSLFGGVAWQWEP----RREQYYLHNF 165
17   KDYYI-----WVDPVKDDKG-NPIKDKYPN-NWLSVFNGTGWTFHE----GRKQFYFHQF 194
35   ADYYI-----WHDCMQTGG-----LVVPPN-NWVSFYGNSAWEYDD----TRKQCYLHQF 280

   CSR V                              CSR II
                                        β4
20   GAAMPKVNTQNPAVQAYL-----IDVTKYWIEQFGIDAWRFDVADEVDHG---------- 342
21   YASLPKLDYQSESLVNEIYRGEDSIVRHWLKAPWNMDGWRLDVVHMLGEAGGARNN---- 350
18   TPQQVDIDTDSDKGWEYL-----MSIFDQMA-ASHVSYIRLDAVGYGAKEAGTSCFMTPK 210
```

```
NI   WSDVKDLDYGHHELWQYQ-----IDTLLYWS-Q-FVDGYRCDVAPLVPLD---------- 179
34   -------------FEDNV-----TEAVNVWLERGVGGIYFGDSE---------------- 250
4    NSFQWDLNYSNPWVFRAM-----AGEMLFLA-NLGVDILRMDAVAFIWKQMGTSCENLP- 311
36   WSGMPDLNFDNPEVRKEM-----IKVGKYWL-QQGADGFRLDAAMHIFRGQTKDGADKN- 256
16   YSHQPDLNYENPKVLAEM-----LDALRFWL-GFGVDGFRLDAVPYLVEEEGTNCE---- 265
29   DVTQADLNWENEEVRKHV-----YDMMHFWF-EKGIDGFRLDVINLISKDQRFPNAEEG- 220
31   SKKQPDLNWDNEKVRQDV-----YEMMKFWL-EKGIDGFRMDVINFISKEEGLPTVETEE 217
30   DVEQPDLNWENPEVRAEF-----ADILRFWL-DRGVDGFRIDVAHGMIKDPALPDIAEGQ 239
23   LVDQPDLNFHNAEVQQAT-----LDNVRFWL-DRGVDGFRLDAINFCFHDAQLRDNPAKP 219
17   YKQQPDLNYRNSDVREEM-----KNIMKFWL-DKGIDGFRIDAVPHLFESANISLDEPP- 247
35   KKEQPDLDFNNPDVNEEI-----LNIIKFWL-EKGVDGFTINSAKFLLEAEHLRDEPQVN 332
                                               .     .

                                                       CSR III
                                                         β5
20   -------------------------------FWRALCGALRAIKPDVYLLGESWHSSQ-- 364
21   -----------------------------MQHVAGITEAAKETQPEAYIVGEH------- 374
18   ----------------------------TFKLISRLREEGVKR------GLE-------- 228
NI   -------------------------------FWLEARKQVNAKYPETLWLAESAGS--GF 206
34   ------------------------NFPNASSFLSEWGNMT-AN------YSKEGKPRVLLL 285
4    ---------------------------QAHALIRAFNAVMRIAAPAVFFKSE------AI 338
36   -----------------------------IAWWNEFRSEMEKVNPNVYLAGE------VW 281
16   ------------------------NLPGTHDILKQVRKMVDEEFPGRILLCE-----ANQ 296
29   ----DGRSFYTDGP-------------RVHEFLHEMNEKVFSHYDS-MTVGEMSS----T 258
31   EGYVSGHKHFMNGP-------------NIHKYLHEMNEEVLSHYD-IMTVGEM----PGV 259
30   KADML------DG--HTRLPYFDQD--GVHEIYREWRAIVDSYPGERALVAE-----AWV 284
23   ADKRVGRGFSADNPYAYQYHYFNNTQPENLPFLERLRGLLDSYPGAVSL-GEISS----E 274
17   LGKNLN-----LSLHASLNHTLTKDQPETYELVKEWRDFVDNYAEENKR-DEIVLLTEAY 301
35   KLQNPDTISNYSELFHDYTTTQVGMHDIIRNFRQTINKYSREPGRYRFMGTESNDQ---- 390

20   -------SLVGN------GQFNAVMNYPL-TQPILALF---N---G---QLNPEDYVGKT 406
21   --FGDARQWLQA------DVEDAAMNYRGFTFPLWGFL---ANTDI---SYDPQQIDAQT 420
18   -ILIEVHSYYKK-QVEIASKVDRVYDFA-----LPPLL---LHALSTGHVE--------- 269
NI   IEELRSQGYTGLSDSELYQAFDMTYDYD-----VFGDFKDYWQGRS-------------T 248
34   STSTVHNNLAGPS----NETVQGTLFYR-----FLDAE---NKK------------NFSS 316
4    VHPDQVVQYIGQ------DECQIGYNPL-----QMALL---WNTLATREV---------N 375
36   DKPETIAPYYGP--------LHSLFNFD-----LGGTI---LNSVKNGRDQGIATFAEKT 325
16   -WPHDVVEYFG-D----NDECQMAFHFP-----VMPRL---YMGVA---RHTR------- 332
29   -TVDHCIRYTNPD----NKELDMTFSFH-----HLKVD---YPNGE---KWALAPFDFLK 302
31   -TTEEAKLYTGEE----RKELQMVFQFE-----HMDLD---SGEGG---KWDVKPCSLLT 303
30   ENAERVARYLRP------DELHQAFNFE-----YLTAD---WDAA--------------T 316
23   DSLATTAEYTAQ------GRLHMGYSFE-----LLVQD---YSAA--------------Y 306
17   SSLENTLKYYEVG-------SNVPFNFK-----FITDA---NSSS-----------TPEQ 335
35   AAVDKTMLYYGNS--FIQEADFPLNA ------LFDLN---KANI-----------DGAS 428
                          .
                            CSR IV
                              β7
20   NLELM-----MYRQ-P-NQQAMFNALDTHDTPR----------LLT-------------- 435
21   CMAWMDNYRAGLSH-Q-QQLRMFNQLDSHDTAR----------FKT-------------- 454
18   ---PVAHWTDIRPNN------AVTVLDTHDGIG----------VIDIGSDQLDRSLKGLV 310
NI   VERYVDLLQRQDATFP-GNYVKMRFLENHDNAR----------MMS-------------- 283
34   LGATIKQYVAET-----GILGNSWMVGAPQMRH----------LAS-------------- 347
4    ---LLHQALTYRHNLPEHTAWVNYVR-SHDDIGWTFADEDAAYLGISGYDHRQFLNRFFV 431
36   LQ-LYKSYN--------KAALDAPFLSNHDQTR----------VMS-------------- 352
16   --EAITTILASTPPVPQGCQWAT-FLRNHDELT----------LEMVTEDDRQYMWREYA 379
29   LKEILSDWQT---GMHAGGGWNALFWCNHDQPR----------VVS-------------- 335
31   LKENLTKWQK---ALE-HTGWNSLYWNNHDQPR----------VVS-------------- 335
30   LRAVVDRSLA---ANNAVGAPTTWVLSNHDVTR----------HVT-------------- 349
23   IRDTVSRLEA-----TMLEGWPCWAISNHDVVR----------AVT-------------- 337
17   FKVIIDNWIKGT---P-QNNVPNWVMGNHDRVR----------VGT-------------- 367
35   IFSMVDLWMKAM---P-SGKWPNWMVGGPSSSR----------IAS-------------- 460
```

```
                                             .

20   -------------------------------------------TLH-------------G 439
21   -------------------------------------------LLG-------------R 458
18   PDEDVDNLVNTIHANTHGESQAATGAAASNLD-LYQVNSTYYSALG-------------C 356
NI   -------------------------------------------L--------------MH 286
34   ----------------------------------------------------------MV 349
4    NRF--------------D------GSFARGVPFQYNPSTGDCRVSGTAAALVGLAQDDPH 471
36   -------------------------------------------ELG-------------G 356
16   P------------------------------------DPRMRLNLGIRRR---LAPLMGN 400
29   -------------------------------------------RYG---------DDGAY 343
31   -------------------------------------------RFG---------NDGMY 343
30   -------------------------------------------RFG---------GGAQ- 356
23   -------------------------------------------RWG---------GAQA- 344
17   -------------------------------------------RYP-------------- 370
35   -------------------------------------------RVG-------------- 463

                     CSR VII
                       β8
20   QLTKFKSALTLLMLLPGSPCIYYGTEVAMAGGAD-------------------------- 473
21   DIARLPLAVVWLFTWPGVPCIYYGDEVGLDGKND-------------------------- 492
18   NDQHYIAARAVQFFLPGVPQVYYVGALAGKNDMELLRKTNN------------------- 397
NI   SKAEAVNNLTWIFMQRGIPLIYNGQEFLAEHQPSLFDRDTM------------------- 327
34   NENLLRVYQLLLFTLPGTPISLYGDEIGLKDLSGLAAQ---------------------- 387
4    AVDRIKLLYSIALSTGGLPLIYLGDEVGTLNDDDWSQDSNK------------------- 512
36   DVRKAKLAASILLTLPGQPFLYYGEEIGMKGEKPD------------------------- 391
16   DPDKIRLMNAMLLSLPGSPVLYYGDEIGMGDDIW-------------------------- 434
29   RVKSAKMLATAIHMMQGTPYIYQGEELGMTNPKFTDISSYRDVESLNMYHAFK-EKGMAD 402
31   RIESAKMLATVLHMMKGTPYIYQGEEIGMTNVRFESIDEYRDIETLNMYKEKVMERGEDI 403
30   GLARARAALLLMLALPGSVYLYQGEELGLPEVTDLPEEALQD-----------------P 399
23   TPAFARMVVALLCSLRGSICLYQGEELGLSEAEV-AFEDLQD-----------------P 386
17   -GR-ADHMIMLEMILPGVAVTYYGEEIGMVDNTTIY--KYD------------------- 407
35   -RQYVNVMNMLLLTLPGTPTTYYGEELGMEDGSPQV--STEN------------------ 502
                     *         .

20   ------------PDNRRCMNWQPDEQ---------------------------------- 487
21   ------------PFCRKPFPWQVEKQ---------------------------------- 506
18   ----------GRDINRHYYS---T------------------------------------ 408
NI   --------VADRHGDVTPLIQKLVTIKQLPLLRAADYQLAVVEEGIVKITYRAAGEALTA 379
34   -------------LSGPIMQWGEVSV--------------------SN---NSP------ 405
4    ---------------SDDSRWAHRPR---------------------------------- 523
36   ------------EYLREPMRWYKGDG-----------------PGQTT------------ 410
16   --------LHDRDGVRTPMQWTADPS-----------------AGFST---ATP---DKF 463
29   QDITAILQAKSRDNSRTPVQWDATEN-----------------GGFTT---GTP------ 436
31   EKVMQSIYIKGRDNARTPMQWDDQNH-----------------AGFTT---GEP------ 437
30   TWKRSGYTERGRDGCRVPLPWEGDEP--------------PFGFGCSA---ERS------ 436
23   YGITFWPTFKGRDGCRTPMPWTDA-P--------------SA--GFTS---GKP------ 420
17   ----------VRDGCRTPFQWDNSIN-----------------AGFSK---IAENLLEKN 437
35   ----PQEYNPTEYPEKTPMQWDSSSN-----------------AGFSE---AN-----RT 533

20   -------------------------EQQVRQFVTRLIKFRRQQADFLATSQLTWTIDGHC 522
21   -------------------------DTALFALYQRMIALRKKSQALR---------HGGC 532
18   ------------------AEIDENLKRPVVKALNALAKFRNELDAFD----------GTF 440
NI   WIPLK------GQ---VTAVATKLAAGSY----QNLLTDGP--TEVV---------DGKL 415
34   ---QI--A--TDVNANVTFKGQDADKGSFLNVYRKLSDLRGKERSLL---------HGDF 449
4    ----------YNEALYAQRNDPSTAAGQIYQGLRHMIAVRQSNPRFD---------GGRL 564
36   WEKP---KYNTGE---VSVEAQLRDDDSLLESYRTLIRLREEHDALR---------SDSL 455
16   FLPLLADPYSPQQ---VNVTDQMADPGSLLVWMRSMLTTRRQFPVFG---------RGEF 511
29   WIPV---AGNYRE---INAEAALRDQNSVFYHYQKLIQIRKMYDIVT---------EGTY 481
31   WITV---NPNYKE---INVKQAIQNKDSIFYYYKKLIELRKNNEIVV---------YGSY 482
```

```
30        WLPV---PAEWRS---LTREVQERDPDSTLSLYKKALRLRRELLLPA---------EDAL 481
23        WLPL---AASHRA---AAVSVQQDDAHSVLRAVRAFLAWRKEMPALR---------EGSI 465
17        WLPV---HTSYKSG--LNLEQEKKDSISHYHLYTNLTALRKRDVLKK----------GNF 482
35        WLSV---NPDYEA---VNVEAQKNEQYSTLNLYRELNHLRNNELPLH---------RGWL 578
                                                                   .

20        LILTR-TDVE--------------QTIQGRFNLG--AQAVSTVSGVSPILT-AGITA--- 561
21        QVLYA-EDNVVVFVRVLNQ-----QRVLVAINRG--EA-CEVVLPASPFLN-AVQWQ--- 579
18        SYTTDD-DTSISFTWRGETS----QA---TLTFE--PKRGLGVDNTTP--VAMLEWED-- 486
NI        TVD--GQPVLIKYVTNTAVT-----KVADQSN---------------------------- 440
34        ALLYNS-DQAISFLRSWDQNE----RYVTALNFNYEGEAELFLKQDGDKEL---PEQGTV 501
4         VTFNTNNKHIIGYIRN--------NALLAFGNFS--EYPQTVTAHTLQAMP----FKAHD 610
36        QPIQAGSASVTAFKRTSDK-----ESLYVYHNLS--GEPVTLQMKDFD----KGKWKVVF 504
16        IDMGGQNDAVLSFLRVLDDDKHGSQRLLCLNNLS--PEEQDVLL-YLPDDAGTKPVS--- 565
29        EIIAKDDPNIFAYLR-----HGSNEKLLVINNFY--GTEAAF---TLPDSLAPDEWKAEV 531
31        DLILENNPSIFAYVR-----TYGVEKLLVIANFT--AEECIF---ELPEDISYSEVE--L 530
30        HWADAP-QNVLAFRR-----EPGFT---CAVNFG--ADPVTL---PFEGEV--------- 518
23        AFYDTA-EPVLMFRR-----EHAGQVVLLAFNLS--ADPAEL---ALPAGE----WEQID 510
17        TIEIL-NKTVLAVVRQSEEE-----AVSLLINFS--KNNTIVDISKLVNKRNNAK----I 530
35        CYTWS-DNNVFAYVRELDGLNK---VFMMVLNFG--QAWTINLRQAVPDLPGEAK----I 628
                                         .

20        --------NE-----MAPGGFELTVA----------------------------------- 574
21        --------CKEGHGQLTDGILALPAISATVWMN--------------------------- 604
18        ---------SAGDHRSDDLIANPPV-VA-------------------------------- 504
NI        ------------------------------------------------------------ 440
34        VLSS-NPQRKDGDTVSLKSL-K-----------LGAGEALLLKYPYSG------------ 536
4         --------LIGGKTVSLNQDLTLQP-YQVMWLEIA------------------------- 636
36        STSK----DV----KVKKGTITIPA-YSSLITKEDRKS---------------------- 533
16        LLTS---QAMAPIAANGEFHDTLPA-WGFAWLDLSKGSDEHESE---------------- 605
29        LLT----NDEARE-GLQNMTLR-PY-ESIVYR-LTKPC---------------------- 561
31        LIHN---YDVENG-PIENITLR-PY-EAMVFK-LK------------------------- 558
30        VLSS---GPVE----QDGTHLVLPG-DTAVWL-RQ------------------------- 544
23        VPGV---ELGA----MDGGHLRLAG-HAVVAA-VGRG----------------------- 538
17        YTSSVNSNLTVNQTVNPVAI-NIPG-DTSIIVDSSTSGATIVN--YS--IMIFLSAVFIS 584
35        RLSTEGSRV--GKAVSTDSIQTQPG-EGL-ILEYKTNKPVHVRDTFKDKCFISEKACYTS 684

20        ---------      574
21        ---------      604
18        ---------      504
NI        ---------      440
34        ---------      536
4         ---------      636
36        ---------      533
16        ---------      605
29        ---------      561
31        ---------      558
30        ---------      544
23        ---------      538
17        FFQR-----      588
35        AFDLLYNSC      693
```

Fig. S1. Sequence alignment of the α-amylase representatives of thirteen different GH13 subfamilies with focus on the intermediary group with MPDLN as CSR V (Majzlová et al., 2013). The sequences from GH13_4 (*Neisseria polysaccharea*, accession Q9ZEU2), GH13_16 (*Propionibacterium freudenreichii* subsp. *Shermanii*, A1XGB1), GH13_17 (*Apis mellifera*, Q25BT8), GH13_18 (*Bifidobacterium adolescentis*, Q84HQ2), GH13_20 (*Lactobacillus plantarum* WCFS1, F9URM8),

GH13_21 (Escherichia coli, P21517), GH13_23 (*Xhantomonas campestris*, Q76LB0), GH_29 (Bacillus subtilis, P39795), GH13_30 (*Thermonospora curvata*, Q60027), GH13_31 (*Bacillus cereus*, P21332), GH13_34 (*Xenopus leavis*, Q7ZYR3), GH13_35 (*Xenopus leavis*, Q32NL8), GH13_36 (*Paenibacillus polymyxa* E681, E0RLH8) subfamilies and Lp_0179 (NI, not included in a subfamily, F9USZ) are represented. Aligment was done using the program ClustalOmega. Residues that are identical (*), conserved (:) or semiconserved (.) in all sequences are indicated. Dashes indicated gaps introduced to maximize similarities. The GH13 three domains are highlighted in blue (domain A), green (domain B), and yellow (domain C). The catalytic triad is highlighted in pink. The seven GH13 CSRs are boxed. Conserved residues in amylolytic enzymes are highlighted in red (Lee et al., 2002)
