# Supplementary material for: Unravelling the diversity of glycoside hydrolase family 13 α-amylases from Lactobacillus plantarum WCFS1
Source: Microb Cell Fact. 2019 Oct 26;18:183. doi: 10.1186/s12934-019-1237-3 (PMC6815381; doi:10.1186/s12934-019-1237-3)
Supplement: Supplementary file 2 — Additional file 2: Figure S2. Sequence alignment of the α-amylase family GH13_20 representatives from Alicyclobacillus acidocaldarius (AAC) (accession Q9WX32), Thermoactinomyces vulgaris (TVU) (Q08751), Klebsiella oxytoca (KOX) (Q48398), Bacillus sp. I-5 (BSP) (Q59226), Lysinibacillus sphaericus (LSP) (Q08341), Anoxybacillus flavithermus (AFL) (Q5BLZ6), Geobacillus caldoxylosilyticus (GCA) (CoLZ63), Thermus sp. IM6501 (O69007), Bacillus stearothermophilus (BST) (P38940), and Lactobacillus plantarum WCFS1 (LPL) (F9URM8). Aligment was done using the program ClustalOmega. Residues that are identical (*), conserved (:) or semiconserved (.) in all sequences are indicated. Dashes indicated gaps introduced to maximize similarities. The GH13 three domains are highlighted in blue (domain A), green (domain B), and yellow (domain C). The N-terminal domains are indicated in dark blue. The seven conserved sequence regions (CSR) found in GH13_20 subfamily α-amylases are also indicated. The residues of the GH13 catalytic triad (Asp-171, Glu-200, and Asp-227 in Lp_0179) are highlighted in pink colour. Conserved residues in amylolytic enzymes are highlighted in red [6]. The conserved VAnE and MPKLn motifs, in the CSR II and V respectively, are indicated in grey colour. [file 12934_2019_1237_MOESM2_ESM.pdf]

Figure S2

```
AAC     ---MELVWQHRWAADAYPLDLSTMVLVLRVARCTPAQRVRVHFGDRYEPDFQ--------    49
LPL     -MQLAGIRHRPESEDTAVLAAHQLQVRLQTARD-DVAQVEILFADSYLWQEGTTA-----    53
KOX     MITISSLSHSAKSADSYAYNKETLHIRFRTVKG-EVAKVSLWIGDPYQWEEGGLDGGNLG    59
TVU     -MLLEAIFHEAKGSYAYPISETQLRVRLRAKKG-DVVRCEVLYADRYASPEEE-------    51
AFL     -MLKEAVYHRPTDQFAYAYDDKTLHIRLRTKKS-DVHAVSLLHGDPYCWEDRQWQ-----    53
GCA     -MFKEAIYHRPKDNFAYAYDEKTLHIRLRTKKD-DVEAVHLLYADPYEWKDGVWQ-----    53
TSP     -MRKEAIHHRSTDNFAYAYDSETLHLRLQTKKN-DVDHVELLFGDPYEWHDGAWQ-----    53
BST     -MRKEAIYHRPADNFAYAYDSETLHLRLRTKKD-DIDRVELLHGDPYDWQNGAWQ-----    53
BSP     -MFLEAVYHRPRKNWSYAYNGTTVHLRIRTKKD-DMTAVYALAGDKYMWDHT--------    50
LSP     MIMLEAVYHRMGQNWSYAYNDSTLHIRIRTKRD-NVPRIDLHCGEKYDPEKY--------    51
              : :      :       : : ::. :           .: *

AAC     ----------NSSEARRYGSDGTFDWFTCRVAAPTRRLKYAFEVLSSTGRAVYLGEGGLA    99
LPL     ---------LERRIMQPGLATQGNQYWQTTLTVPTNRVVYAFLITDTIGATVGYGEGGFF    104
KOX     GSDAHGWSGGNEVHMEKEGQSESYDHWFAAFTPCKRRSRYGFILYGNNGEKLLFGERRCV    119
TVU     ---------LAHALAGKAGSDERFDYFEALLECSTKRVKYVFLLTGPQGEAVYFGETGFS    102
AFL     ---------FTKTEMTKSGSDALFDYWWVAISPPYRRLRYGFELVSGN-ERCVYTEKGFF    103
GCA     ---------LNRAPMVKSGSDDLFDYWFIEVIPPYRRLRYGFELTSGN-EKIVYTEKGFY    103
TSP     ---------FQTMPMRKTGSDGLFDYWLAEVKPPYRRLRYGFVLRAGG-EKLVYTEKGFY    103
BST     ---------FQMMPMRKTGSDELFDYWFAEVKPPYRRLRYGFVLYSGE-EKLVYTEKGFY    103
BSP     ---------MEYVPMTKLATDELFDYWECEVTPPYRRVKYGFLLQQGH-EKRWMTEYDFL    100
LSP     ---------KETIPMERMASDGLFDYWQAAVQPRYRRLVYYFALHSDNGDAVYFMEKGFF    102
                                : :   .    .*  * * :           *

AAC     DTL----------DAVQPFQYPYIHPSRVLAVPDWVGHAVAYQIFPDRFAVGEQQLV-RP    148
LPL     DDV-----ATNWQTVGNYFHMPYMHVSDAELPPAWVKETVWYQIFPERFANGVRQNDPAN    159
KOX     DISKPPVAETELSNLSNFFCFPYINPGDVLSTPEWVKKTIWYQIFPERFCNGDPSISPEN    179
TVU     A-------ERS---KAGVFQYAYIHRSEVFTTPEWAKEAVIYQIFPERFANGDPSNDPPG    152
AFL     H-------EAPIDDIAYYFCFPFLNRIDVFHAPTWVKDTVWYQIFPERFANGDPSLNPKG    156
GCA     H-------EAPTDDTAYYFCFPFLNKIDVFHAPEWVKDTVWYQIFPERFANGNPAIDPEG    156
TSP     H-------EAPSDDTAYYFCFPFLHRVDLFQAPDWVKDTVWYQIFPERFANGNPAISPKG    156
BST     F-------EVPTDDTAYYFCFPFLHRVDLFEAPDWVKDTVWYQIFPERFANGNPSISPEG    156
BSP     T-------EPPR-NPDRLFEYPFINPVDVFQPPAWVKDAIFYQIFPERFANGDTRNDPEG    152
LSP     D-------QPPKVMYEGLFDFPYLNRQDVHTPPAWVKEAIFYQIFPERFANGDPSNDPEG    155
                          *   :::        * *. .:: *****:**. *
                                          CSR VI
                                            β2
AAC     TDPWDAR--PTPDSVFGGNLRGIVDKLPYLSDLGVNLMYLTPIFQAPSNHKYDTQDYFAV    206
LPL     VQPWGAG-RVHRDSFYGGDLPGITAHLDDLAALGVNGLYLCPIFTSPSNHKYDTIDHFEI    218
KOX     VQPWGTP--PDSKNFMGGDLQGIINKLDYLQDLGVNGLYLCPIFTANASHKYDTVDYFNV    237
TVU     TEQWAKDARPRHDSFYGGDLKGVIDRLPYLEELGVTALYFTPIFASPSHHKYDTADYLAI    212
AFL     TIPWGSA-DPTPTSFFGGDFAGIMERLDYLVELGINGIYFTPIFKASSNHKYDTIDYFEI    215
GCA     TLPWASE-DPTPTSFFGGDLEGIIQHLDYLVDLGVNGIYLTPIFHAPSNHKYDTTNYFEI    215
TSP     ARPWGSE-DPTPTSFFGGDLQGIIDHLDYLADLGITGIYLTPIFRAPSNHKYDTADYFEI    215
BST     SRPWGSE-DPTPTSFFGGDLQGIIDHLDYLVDLGITGIYLTPIFRSPSNHKYDTADYFEV    215
BSP     TLPWGSA-DPTPSCFFGGDLQGVIDHLDHLSKLGVNAVYFTPLFKATTNHKYDTEDYFQI    211
LSP     VQEWGGT--PSAGNFFGGDLQGVIDHLDYLSDLGVNALYFNPLFAATTNHKYDTADYMKI    213
           *          . **:: *:  :*  *  **:. :*: *:* : : ***** ::: :
                       CSR I
                         β3
AAC     DPAFGTLGDLQLLVREAHRLGIRVVLDAVFNHSGFQFAPFQDVIARGTASPYWSWFFVQG    266
LPL     DPHFGTKADFQALVDGAHARGMRVMLDAVFNHFGEQSPQWQDVIKNGEQSRFADWFHIHG    278
KOX     DPHFGGNDRFKELVQKAHQRGMKVMLDAVFNHIGNQSPLWLDVVKNGDKSPYADWFWIKK    297
TVU     DPQFGDLPTFRRLVDEAHRRGIKIILDAVFNHAGDQFFAFRDVLQKGEQSRYKDWFFIED    272
AFL     DPQFGDKPTFKRLVERCHQKGIRVMLDAVFNHSGHFFAPFQDVLKHGEKSKYKDWFHIHE    275
GCA     DPHFGDKQTFKKLVDLCHEKGIRVMLDAVFNHCGYYFAPFQDVLKNGEASKYKDWFHIHE    275
TSP     DPHFGDKETLKTLVKRCHEKGIRVMLDAVFNHCGYEFAPFQDVLKNGAASRYKDWFHIRE    275
BST     DPHFGDKETLKTLIDRCHEKGIRVMLDAVFNHCGYEFAPFQDVWKNGESSKYKDWFHIHE    275
```

```
BSP       DPQFGDKDTLKKLVDLCHERGIRVLLDAVFNHSGRTFPPFVDVLKNGEKSKYKDWFHIRS    271
LSP       DPQFGTNEKLKELVDACHARGMRVLLDAVFNHCGHTFPPFVDVLNNGLNSRYADWFHVRE    273
          ** **    :: *:  .*  *::::******* *     : **  .*  * : .** :.
                                      CSR V                     CSR II
                                                                  β4
AAC       DRVDV---------ESVNYETFATRLRHMPKLNLAEPAAEEYFLQVAKHYVLECDIDGWR    317
LPL       WPVGR-DPK----TKRLNYETFAT-GAAMPKVNTQNPAVQAYLIDVTKYWIEQFGIDAWR    332
KOX       FPVYPSGDKNEWDFRNFNYETFGN-VIEMPKLNTENPACRDYLLQVARYWIEEFDIDGWR    356
TVU       FPVSK-TSR-------TNYETFAVQVPAMPKLRTENPEVKEYLFDVARFWME-QGIDGWR    323
AFL       FPLQT-EPL-------PNYDTFAF-VPTMPKLNTENQEVKQYLLDVATYWIREFDIDGWR    326
GCA       FPLQT-TPR-------PNYDAFAF-VAQMPKLNTENPEVKKYLLDVAAYWIQEFDIDGWR    326
TSP       FPLQT-EPR-------PNYDTFAF-VPHMPKLNTAHPEVKRYLLDVATYWIREFDIDGWR    326
BST       FPLQT-EPR-------PNYDTFRF-VPQMPKLNTANPEVKRYLLDVATYWIREFDIDGWR    326
BSP       LPLEVVDGI-------PTYDTFAF-EPLMPKLNTEHPDVKEYLLKAAEYWIRETGIDGWR    323
LSP       WPLRVVDGI-------PTYDTFAF-EPIMPKLNTGNEEVKAYLLNVGRYWLEEMGLDGWR    325
            :              .*::*      ***:.  .   .  *::..  .::   .:*.**
                                      CSR III
                                        β5
AAC       FDVANEIDPHFWSRLRSELRALKPDILLIGEIWHDSLPWLMGDAFDGVMNYPLRELVMRY    377
LPL       FDVADEVDHGFWRALCGALRAIKPDVYLLGESWHSSQSLVGNGQFNAVMNYPLTQPILAL    392
KOX       LDVANEVDHEFWRAFRRTVKSIKPDCYILGEIWHEGMPWLRGDQFDSLMNYPLMQATTDY    416
TVU       LDVANEVDHAFWREFRRLVKSLNPDALIVGEIWHDASGWLMGDQFDSVMNYLFRESVIRF    383
AFL       LDVANEVDHQFWREFRQAVKAVKQDVYILGEIWHDAMPWLRGDQFDAVMNYPFTNGVLRY    386
GCA       LDVANEIDHQFWREFRQVVKSMKPDVYILGEIWHDAMPWLRGDQFDAVMNYPFTNGAIRF    386
TSP       LDVANEIDHQFWREFRQAVKALKPDVYILGEIWHDAMPWLRGDQFDAVMNYPLADAALRF    386
BST       LDVANEIDHEFWREFRQEVKALKPDVYILGEIWHDAMPWLRGDQFDAVMNYPFTDGVLRF    386
BSP       LDVANEVSHQFWREFRRVVKQANPDAYILGEVWHESSIWLEGDQFDAVMNYPFTNAVLDF    383
LSP       LDVANEVDHQFWREFRSEIKRINPSAYILGEIMHDSMPWLQGDQFDAVMNYPFTNILLNF    385
          :***:*:.  **  :   ::  : .  ::**  *..   : .. *:.:*** : :
                                          CSR IV
                                            β7
AAC       AMDESIDEPAFAEAWVRLYLQYPRPAWRAMWNLLGSHDTERALTRARGHVPSVVLAFAML    437
LPL       FNGQ-LNPEDYVGKTNLELMMYRQPNQQAMFNALDTHDTPRLLTTLHGQLTKFKSALTLL    451
KOX       FALQAYDKKTFIDIVTHAYLCYPRNVNEVMFNLLESHDTSRLLSLCGNDKRKARLAYLFM    476
TVU       FATGEIHAERFDAELTRARMLYPEQAAQGLWNLLDSHDTERFLTSCGGNEAKFRLAVLFQ    443
AFL       FAKSEMKASEFAHSITNVLHSYPNNVNEVAFHLLGSHDTPRILTECGENKEKVKLMFTFQ    446
GCA       FAKNEIGAKQFANIITNVLHSYPENVNEVAFHLLGSHDTPRILTVCDNDVRKVKLLFLFQ    446
TSP       FAKEDMSASEFADRLMHVLHSYPKQVNEAAFNLLGSHDTPRLLTVCGGDVRKVKLLFLFQ    446
BST       FAKEEISARQFANQMMHVLHSYPNNVNEAAFNLLGSHDTSRILTVCGGDIRKVKLLFLFQ    446
BSP       FIHQIADAEKFSFMLGKQLAGYPRQASEVMFNLLDSHDTARLLTQADGDKRKMKLAVLFQ    443
LSP       FARRLTNAAEFAQAIGTQLAGYPQQVTEVSFNLLGSHDTTRLLTLCSGNVERMKLATLFQ    445
                    :          * .   .  :: * :*** * *:    .         :
                CSR VII
                  β8
AAC       FTLPGIPMVYYGDEIGMEGGTD---PDCRRGMIWDANLWQLDLREAVRQLAWLKRTHPAL    494
LPL       MLLPGSPCIYYGTEVAMAGGAD---PDNRRCMNWQPDEQEQQVRQFVTRLIKFRRQQADF    508
KOX       FSQVGSPCIYYGSEVGMNGSRAMGSEDNRKCMIWDEQKQDLEFKSFIKDLILWRKKHSEW    536
TVU       MTYLGTPLIYYGDEIGMAGATD---PDCRRPMIWEEKEQNRGLFEFYKELIRLRHRLASL    500
AFL       FSFTGTPCIYYGDEIGLTGGQD---PLCRKCMVWEEEKQDLELFAHVQKLISLRRKYRSL    503
GCA       LSFTGSPCIYYGDEIGMTGGQD---PGCRKCMVWEEEKQNRDILEHVKKLIALRKAHPAF    503
TSP       LTFTGSPCIYYGDEIGMTGGND---PECRKCMVWDPEKQNKELYEHVKQLIALRKQYRAL    503
BST       LTFTGSPCIYYGDEIGMTGGND---PECRKCMVWDPMQQNKELHQHVKQLIALRKQYRSL    503
BSP       FTYFGTPCIYYGDEVGLDGGHD---PGCRKCMEWDETKHDKDLFAFYQTVIRLRQAHAAL    500
LSP       LTYQGTPCIYYGDEIGMDGEYD---PLNRKCMEWDKSKQNTELLAFFRSMISLRKAHPAL    502
          :   * * :*** *:.: *         *: * *:    :  .      :   ::

AAC       AGDTMEIRDANS--GVLHYVRLNGTGPHLHIAVCKRRDFGAEVDSPLFAWDVPSRTSGEE    552
LPL       LATSQLTWTIDGHC--LILTRTDVEQTIQGR--FNLGAQAVSTVS---------------    549
KOX       NDPKIHWNNV-DHPAVVAFSR----GEVHFL--LNNSDESLAINYQG-------------    576
TVU       TRGNVRSWHADKQANLYAFVRTVQDQHVGVV--LNNRGEKQTVLLQVP-------ESGG-    550
AFL       SRGELHFLETNDETNHLVYTKTYEQETVIFL--LNNSNRKLDITLPLS--------VKR-    552
```

```
GCA      RRGKITFMDASDESNHLIYTKTYLDETILVM--INNSSKEIDITVPLS--------LKG-       552
TSP      RRGDVAFLTADDEVNHLVYAKTDGNETVMII--INRSNEAAEIPMPID--------ARG-       552
BST      RRGEISFLHADDEMNYLIYKKTDGDETVLVI--INRSDQKADIPIPLD--------ARG-       552
BSP      RTGTFKFLTAEKNSRQIAYLREDDQDTILVV--MNNDKAGHTLRCLSG--------M---       547
LSP      RGSGLRFLPVLEHPQLLVYERWDDNERFLIM--LNNEDAPVNVVIPAA--------QPG-       551
                             :             :

AAC      RKILIWECDSTEGCGHDS--------RADGFHNQ---------  578
LPL      -----------GVSPILTAGITANEMAPGGFELTVA-------  574
KOX      ---------------------RTLSLTPFGFYIIGLDHKDIIS  598
TVU      ---KTWLDCLTGEEVHGKQGQLKLTLRPYQGMILWNGR-----  585
AFL      ---KKLTDLWTNKQFVSEENEWTVTLAPYGFLMFLVE------  586
GCA      ---KWLVNLWTNEQFAAEAETLRAAIPPYS-------------  579
TSP      ---KWLVNLLTGERFAAEAETLCVSLPPYGFVLYAVESW----  588
BST      ---TWLVNLLTGERFAAEAETLCTSLPPYGFVLYAIEHW----  588
BSP      --------HSGPICGTTMS------------------------  558
LSP      ---ASWRTVNGEPCAVVEESSIQAALPPYGYAILHAPIAGTAE  591
```

Fig. S2. Sequence alignment of the α-amylase family GH13_20 representatives from *Alicyclobacillus acidocaldarius* (AAC) (accession Q9WX32), *Thermoactinomyces vulgaris* (TVU) (Q08751), *Klebsiella oxytoca* (KOX) (Q48398), *Bacillus* sp. I-5 (BSP) (Q59226), *Lysinibacillus sphaericus* (LSP) (Q08341), *Anoxybacillus flavithermus* (AFL) (Q5BLZ6), *Geobacillus caldoxylosilyticus* (GCA) (CoLZ63), *Thermus* sp. IM6501 (O69007), *Bacillus stearothermophilus* (BST) (P38940), and *Lactobacillus plantarum* WCFS1 (LPL) (F9URM8). Aligment was done using the program ClustalOmega. Residues that are identical (*), conserved (:) or semiconserved (.) in all sequences are indicated. Dashes indicated gaps introduced to maximize similarities. The GH13 three domains are highlighted in blue (domain A), green (domain B), and yellow (domain C). The N-terminal domains are indicated in dark blue. The seven conserved sequence regions (CSR) found in GH13_20 subfamily α-amylases are also indicated. The residues of the GH13 catalytic triad (Asp-171, Glu-200, and Asp-227 in Lp_0179) are highlighted in pink colour. Conserved residues in amylolytic enzymes are highlighted in red (Lee et al., 2002). The conserved VAnE and MPKLn motifs, in the CSR II and V respectively, are indicated in grey colour.
