# Supplementary material for: Unravelling the diversity of glycoside hydrolase family 13 α-amylases from Lactobacillus plantarum WCFS1
Source: Microb Cell Fact. 2019 Oct 26;18:183. doi: 10.1186/s12934-019-1237-3 (PMC6815381; doi:10.1186/s12934-019-1237-3)
Supplement: Supplementary file 3 — Additional file 3: Table S1. Concentrations (mg/mL) of the products after the enzymatic hydrolysis with Lp_2757. [file 12934_2019_1237_MOESM3_ESM.pdf]

Table S1. Concentrations (mg/mL) of the products after the enzymatic hydrolysis with Lp_2757

		**mg/mL reaction**	
**Substrate**	**Products**	**24 h**	**48 h**
Starch	Glucose	1.5	2.1
	Maltose	4.7	6.1
	Maltotriose	0.4	0.3
Amylose	Glucose	2.7	3.6
	Maltose	8.9	10.7
	Maltotriose	0.8	0.5
	Maltotetraose	0.3	0.3
Amylopectin	Glucose	0.1	0.2
	Maltose	0.8	1.2
Pullulans	Glucose	0.2	0.2
	Maltose	0.4	2.5
	Maltotriose	0.5	0.4
	Maltotetraose	0.3	1.7
α-Cyclodextrin	Glucose	5.9	11.4
	Maltose	30.4	36.8
	Maltotriose	14.7	12.3
	Maltotetraose	4.2	5.4
β- Cyclodextrin	Glucose	2.5	9.0
	Maltose	13.4	26.1
	Maltotriose	30.8	8.9
	Maltotetraose	7.5	3.0
γ-Cyclodextrin	Glucose	6.9	11.2
	Maltose	23.5	28.9
	Maltotriose	10.3	8.2
	Maltotetraose	3.6	5.6
Acarbose	Glucose	2.1	3.3
	Maltose	1.3	1.8
	Maltotriose	3.9	5.9
Panose	Glucose	0.2	0.2
	Maltose	0.3	0.4
Maltopentaose	Glucose	26.0	30.8
	Maltose	157.4	160.4
	Maltotriose	26.5	12.5
	Maltotetraose	4.5	2.8
Dextrin	Glucose	16.0	15.2
	Maltose	65.0	58.0
	Maltotriose	6.5	2.6
	Maltotetraose	5.1	4.8
